# Systematic review and meta-analysis of the efficacy and safety of novel monoclonal antibodies for treatment of relapsed/refractory multiple myeloma

**DOI:** 10.18632/oncotarget.16987

**Published:** 2017-04-09

**Authors:** Tiantian Zhang, Sen Wang, Tengfei Lin, Jingmei Xie, Lina Zhao, Zhuoru Liang, Yangqiu Li, Jie Jiang

**Affiliations:** ^1^ College of Pharmacy, Jinan University, Guangzhou 510632, People’s Republic of China; ^2^ College of Food Science & Nutritional Engineering, China Agricultural University, Beijing 100083, People’s Republic of China; ^3^ Department of Hematology, First Affiliated Hospital, Jinan University, Guangzhou 510632, People’s Republic of China; ^4^ Institute of Hematology, School of Medicine, Jinan University, Guangzhou 510632, People’s Republic of China; ^5^ Institute of Dongguan, Jinan University, Dongguan 523808, People’s Republic of China

**Keywords:** monoclonal antibody, elotuzumab, daratumumab, relapsed or refractory, multiple myeloma

## Abstract

Although two newly launched monoclonal antibodies (mAbs), elotuzumab and daratumumab, performed well in patients with relapsed or relapsed/refractory multiple myeloma (RRMM), their efficacy and safety remain uncertain. We therefore performed a systematic review and meta-analysis of the most recent clinical trials that evaluated elotuzumab and/or daratumumab for the treatment of patients with RRMM. Our meta-analysis included 13 clinical trials with 2,402 patients participating. The overall response rate (ORR) was 57% (95% confidence interval [CI]: 38-76%), and the at least very good partial response rate (VGPR) was 32% (95% CI: 19-46%). mAb-based regimens prolonged progression-free survival (PFS, hazard ratio: 0.52, 95% CI: 0.36-0.75) compared to non-mAb-based regimens. Additionally, the efficacy of triplet regimens was superior to that of single or doublet regimens. The same trend was observed in a subgroup analysis of daratumumab and elotuzumab. The most common grade 3/4 adverse events included neutropenia, lymphopenia, thrombocytopenia, anemia, leukopenia, pneumonia, and fatigue. Elotuzumab and daratumumab improved the ORR, at least VGPR, and PFS compared to non-mAb-based regimens. In a pooled analysis, both mAbs had promising efficacy and safety profiles, particularly in triplet regimens. The same trend was observed in daratumumab- and elotuzumab-based regimens. Daratumumab triplet therapy (daratumumab, lenalidomide, and dexamethasone) was superior to other triplet regimens for the treatment of RRMM, and daratumumab monotherapy was more effective than either single agent in heavily pretreated MM patients, suggesting CD38 is an effective target for treatment of RRMM. Additional clinical studies of elotuzumab and daratumumab will be required to validate these results.

## INTRODUCTION

Multiple myeloma is the second most common hematological malignancy. It is characterized by the proliferative disorder of plasma cells in the bone marrow with excessive monoclonal protein production [1]. There were approximately 30,330 new cases of multiple myeloma and 12,650 multiple myeloma-related deaths in the United States in 2016 [2]. Multiple myeloma accounts for approximately 1.8% of all cancers and 15% of all hematological malignancies in the United States [2]. Relapsed multiple myeloma is defined as previously treated multiple myeloma that has progressed and requires salvage therapy. Relapsed/refractory multiple myeloma (RRMM) is defined as disease that is nonresponsive to salvage therapy, or that progresses within 60 days of the last treatment in patients who have achieved a minimal response or better on prior therapy [3]. Standard treatment regimens for RRMM include proteasome inhibitors (PIs) such as bortezomib, and immunomodulatory drugs (IMiDs) such as lenalidomide alone or in combination with glucocorticoids (Table 1) [4]. Although these treatment regimens have improved patient survival, most patients eventually relapse following several lines of treatment [5]. Patients who are refractory to PIs and IMiDs, or who have received at least three prior lines of therapy (including a PI and an IMiD) are defined as heavily pretreated MM patients with highly refractory disease [6].

**Table 1:** Traditional and novel agents for RRMM

Agent	Category	Target point	Approval year by FDA
**Traditional agents**			
Lenalidomide	IMiD	NA	2005
Bortezomib	PI	26S proteasome	2003
Dexamethasone	Glucocorticoid	NA	NA
**Novel agents**			
Elotuzumab	mAb	SLAMF7	2015
Daratumumab	mAb	CD38	2015
Panobinostat	HDAC- inhibitor	HDAC	2015
Ixazomib	PI	Peptide boronic acid proteasome	2015
Carfilzomib	PI	Epoxyketone proteasome	2013
Pomalidomide	IMiD	NA	2013

IMiD: immunomodulatory; PI: proteasome inhibitor; mAb: monoclonal antibody; HDAC- inhibitor: histone deacetylase inhibitor; SLAMF7: signaling lymphocytic activation molecule F7; NA: not available or no target points.

The Food and Drug Administration (FDA) approved pomalidomide (an IMiD) and carfilzomib (a PI) in 2013, which expanded the therapeutic options for multiple myeloma [7, 8]. Four additional drugs were approved in 2015: panobinostat, a histone deacetylase inhibitor [9], ixazomib (an oral PI) [10], elotuzumab (a monoclonal antibody [mAb] that targets the signaling lymphocytic activation molecule F7 [SLAMF7]) [11], and daratumumab (a mAb that targets CD38) [12]. These newly launched drugs are referred to as novel agents hereafter. The results of three recently published phase III randomized controlled trials (RCTs) have demonstrated that elotuzumab and daratumumab are promising therapeutics for multiple myeloma [5, 11, 13]. mAbs have been conjugated to various drugs, toxins, and radioisotopes, which improves efficacy. mAbs against interleukin-6, B-cell activating factor CD138, Dickkopf 1 (DKK1), receptor activator of nuclear factor-κB ligand (RANKL), SLAMF7, and CD38 are currently in clinical development for the treatment of multiple myeloma [14]. Daratumumab is a human IgG1k mAb that targets CD38, a cell surface protein that is highly expressed on myeloma cells and is involved in tumorigenesis, and promotes multiple myeloma cell cytotoxicity [15–17], phagocytosis [17, 18], induces apoptosis [17, 19], and depletes CD38-positive immune regulatory T, B, and myeloid-derived suppressor cells [20]. Elotuzumab is a humanized IgG1 mAb that binds specifically to SLAMF7, also called CS1 (cell surface glycoprotein CD2 subset 1), which is highly expressed on the surface of multiple myeloma cells and on subsets of immune cells including natural killer (NK) cells, NK-like T cells, and a subset of CD8-positive T cells [21]. Elotuzumab promotes activation of NK cells and promotes cytotoxicity through the CD16 pathway [22]. Although both daratumumab and elotuzumab were approved by the FDA through the accelerated track, there is limited clinical data regarding the efficacy and toxicity of single or combination daratumumab- and elotuzumab-based regimens. However, several studies have provided evidence for the efficacy and safety of novel agents such as panobinostat [23], carfilzomib [24], and pomalidomide [24], as well as traditional agents including lenalidomide [25] and bortezomib [26], for RRMM treatment. Therefore, we performed a meta-analysis of RRMM patients treated with elotuzumab or daratumumab to evaluate the efficacy and safety of daratumumab and elotuzumab for RRMM treatment, and provide recommendations for the application of these agents in clinical practice. Additionally, we compared the efficacy and toxicity of daratumumab to other therapeutics in order to determine when it should be applied in clinical practice.

## RESULTS

### Trial selection

**Figure 1:**
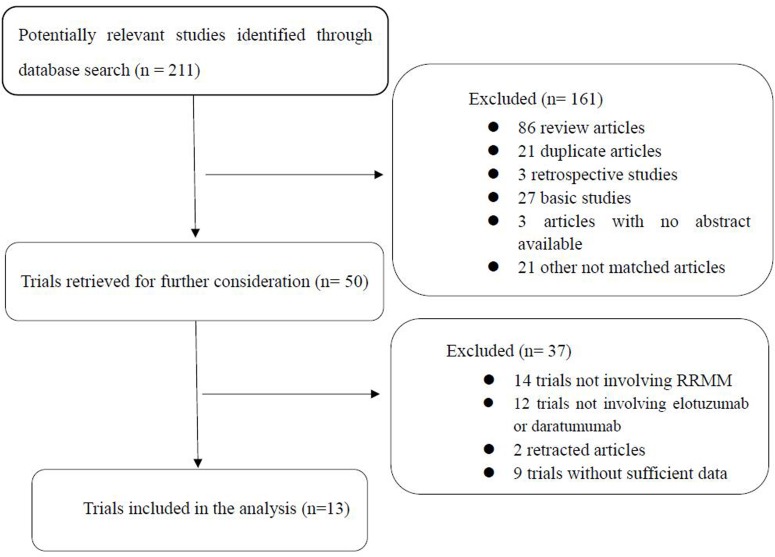
Identification and selection of the studies included in the meta-analysis

The process by which eligible trials were identified and selected for inclusion in our meta-analysis is illustrated in Figure 1. We identified 211 potentially relevant studies in our initial search of several databases. During the screening process, we excluded 161 articles, which included review articles, duplicate articles, retrospective studies, basic studies, and studies with no abstracts available. An additional 37 studies were excluded because they were trials that did not involve RRMM, elotuzumab or daratumumab, were retracted, or did not have sufficient trial data. The final meta-analysis consisted of 13 trials (2,402 patients) that evaluated elotuzumab [11, 27–32] or daratumumab [5, 12, 13, 33–35] for the treatment of RRMM.

### Trial characteristics

All 13 studies included in the meta-analysis were clinical trials that enrolled a total of 2,402 patients. The clinical trial characteristics are shown in Table 2.

**Table 2:** Clinical trials information

Study	Trial name	Phase	Median age (range)	Median prior therapy (range)	No. of patients	Regimen	Dose(mg/kg)	Follow-up (months)	Median PFS time (months)	OS rate -year
Elotuzumab										
Jakubowiak A(2016) [32]	NCT01478048	II	65(25-82)	1(1-3)	77	EVd	10	15.9	9.7	73%-2
			65(25-85)	1(1-3)	75	Vd		11.7	6.9	66%-2
Lonial S (2015) [11]	ELOQUENT-2	III	67(37-88)	2(1-4)	321	ERd	10	24.5	19.4	-
			66(38-91)	2(1-4)	325	Rd		24.5	14.9	-
RichardsonP G(2015) [27]	1703	II	60.6(39-77)	2(1-3)	36	ERd	10	21.2	32.49	-
			63.3(41-82)	2(1-3)	37	ERd	20	16.8	25	-
Mateos(2016) [31]	NCT01632150	II	64(49-82)	3(1-8)	40	ETd	10	-	3.9	63%-1
Jakubowiak A J(2012) [30]	NCT00726869	I	63(41-77)	2(1-3)	28	EV	2.5-20	-	9.46(TTP)	-
Lonial S(2012) [28]	NCT00742560	Ib	60(41-83)	3(1-10)	29	ERd	5,10,20	16.4	NR(TTP)	-
Zonder J A(2012) [29]	NCT00425347	I	64.5(46-87)	4.5(2-10)	35	E	0.5-20	-	-	-
Daratumumab										
Palumbo A(2016) [5]	CASTOR	III	64(30-88)	2(1-9)	240	DVd	16	7.4	NR	-
			64(33-85)	2(1-10)	234	Vd		7.4	7.2	-
Dimopoulos M A(2016) [13]	POLLUX	III	65(34-89)	1(1-11)	286	DRd	16	13.5	NR	92%-1
			65(42-87)	1(1-8)	283	Rd		13.5	18.4	87%-1
Lokhorst H M(2015) [35]	GEN501	II	59(38-76)	4(3-10)	30	D	8	16.9	2.4	77%-1
		II	64(44-76)	4(2-12)	42	D	16	10.2	5.6	77%-1
		I	61.5(42-76)	6.3(2-12)	32	D	0.005-24	-	-	-
Lonial S(2016) [12]	SIRIUS	II	63.5(31-84)	5(2-14)	106	D	16	9.3	3.7	65%-1
Plesner T(2016) [34]	GEN503	II	59.5(41-76)	2(1-3)	32	DRd	16	15.6	NE	90%-1.5
		I	62(48-76)	3(2-4)	13	DRd	2-16	23.5	-	-
Chari A(2015) [33]	NCT01998971	Ib	64(35-86)	3.5(2-10)	77	DPd	16	2.4	-	-

E: elotuzumab; EVd: elotuzumab, bortezomib, and dexamethasone; Vd: bortezomib, dexamethasone; ERd: elotuzumab, lenalidomide, and dexamethasone; Rd: lenalidomide, dexamethasone; D: daratumumab; DRd: daratumumab, lenalidomide, and dexamethasone; DVd: daratumumab, bortezomib, and dexamethasone; DPd: daratumumab, pomalidomide, and dexamethasone; TTP: time to progression; NR: not reached.

### Efficacy

#### Efficacy of mAb-based regimens

**Figure 2:**
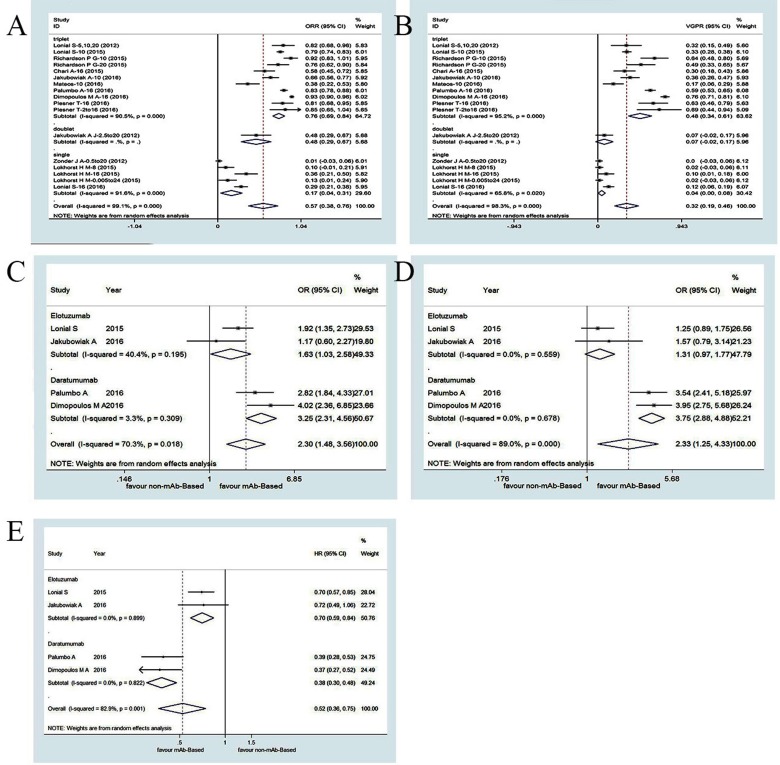
Meta-analysis of the efficacy of mAbs-based regimens in patients with RRMM: (A) overall response rate of mAbs-based single, doublet and triplet regimens;(B) at least very good partial response of mAb-based single, doublet and triplet regimens;(C) odds ratio of overall response of mAb-based triplet compared with controlled arm; (D) odds ratio of at least very good partial response of mAbs-based triplet compared with controlled arm;(E) hazard ratios for progression free survival of mAbs-based triplet compared with controlled arm ORR, overall response rate; VGPR, very good partial response; OR, odds ratio; HR, hazard ratio; CI, confidence interval

All 13 studies (1,472 patients) included in our meta-analysis evaluated the efficacy of elotuzumab or daratumumab for RRMM according to the overall response rate (ORR) and the rate of achievement of at least very good partial response (VGPR). The ORR was 57% (95% confidence interval [CI]: 38−76%) and the at least VGPR rate was 32% (95% CI: 19−46%) (Figure 2A, 2B). In a subgroup analysis (triplet/doublet/single regimen), the ORR was 76% (95% CI: 69−84%) for triplet regimens, suggesting they were more effective than doublet regimens (48%, 95% CI: 29−67%). The doublet regimens were more efficacious than single regimens (17%, 95% CI: 4−31%). The same trend was observed in the at least VGPR. The at least VGPR was 48% (95% CI: 34−61%) for triplet regimens, 7% (95% CI: -2−17%) for doublet regimens, and 4% (95% CI: 0−8%) for single regimens, indicating the triplet regimens were the most effective.

We next analyzed four head-to-head RCTs consisting of 1,865 RRMM patients that compared - to non-mAb-based triplet regimens (Figure 2C-2E) [5, 11, 13, 32]. The overall weighted odds ratios [ORs] of the ORR and at least VGPR were 2.30 (95% CI: 1.48−3.56) and 2.33 (95% CI: 1.25−4.33), respectively, which suggested that mAb-based regimens were more favorable. The overall progression-free survival (PFS) weighted hazard ratio [HR] was 0.52 (95% CI: 0.36−0.75), which also indicated mAb-based regimens were more effective than non-mAb regimens. Thus, although all mAb-based regimens (triplet/doublet/single) displayed impressive efficacy profiles, mAb-based triplet regimens were superior to doublet and single regimens based on the ORR and at least VGPR. In addition, mAb-based triplet regimens were superior to non-mAb-based regimens based on the ORR, at least VGPR, and PFS.

#### Efficacy of elotuzumab-based regimens

**Figure 3:**
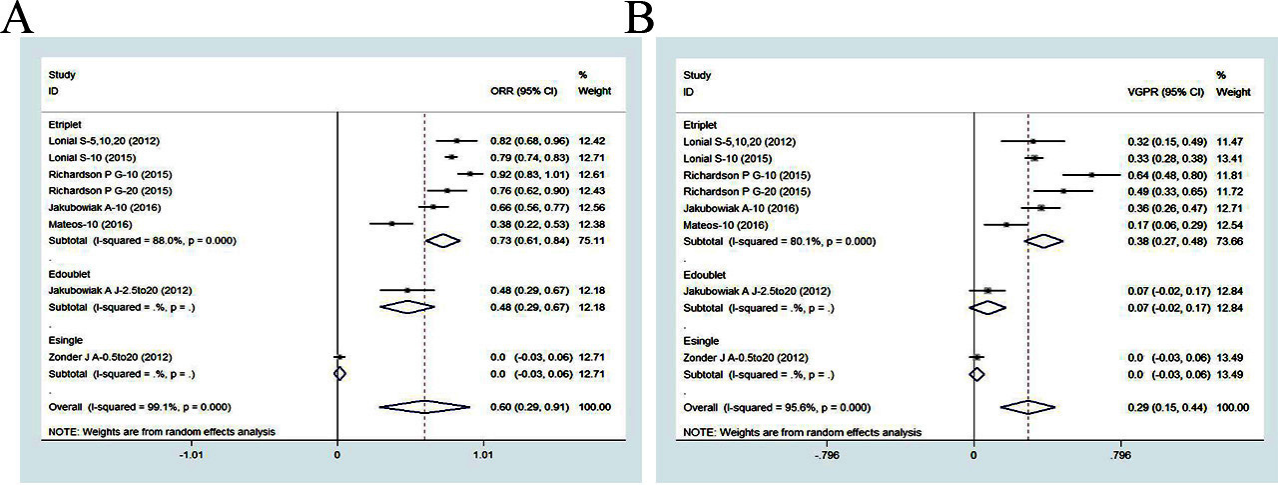
Meta-analysis of the efficacy of elotuzumab-based regimens in patients with RRMM: (A) overall response rate of elotuzuamb-based single, doublet and triplet regimens; (B) at least very good partial response of elotuzumab-based single, doublet and triplet regimens ORR, overall response rate; VGPR, very good partial response; CI, confidence interval. Etriplet, elotuzumab-based triplet regimen; Edoublet, elotuzumab-based doublet regimen; Esingle, elotuzumab-based single regimen

There were seven studies (603 patients) that evaluated the efficacy of elotuzumab for the treatment of RRMM based on ORR and at least VGPR [11, 27–32]. The ORR was 60% (95% CI: 29−91%) and the at least VGPR was 29% (95% CI: 15−44%) in a pooled analysis (Figure 3A-3B). In a subgroup analysis (triplet/doublet/single), the ORR was 73% (95% CI: 61−84%) for triplet regimens, 48% (95% CI: 29−67%) for doublet regimens, and 0% (95% CI: -3−6%) for single regimens, which failed to elicit a significant response in RRMM patients. These results indicated that triplet regimens were superior to doublet and single regimens. The same trend was observed in the at least VGPR. The at least VGPR was 38% (95% CI: 27−48%) for triplet regimens, 7% (95% CI: -2−17%) for doublet regimens, and 0% (95% CI: -3−6%) for single regimens, also suggesting that triplet regimens were the most efficacious.

We analyzed the ORs and HRs reported in two head-to-head RTCs (798 RRMM patients) that compared elotuzumab-based triplet regimens versus non-elotuzumab-based regimens (Figure 2C-2E) [11, 32]. The overall weighted ORs for the ORR and at least VGPR were 1.63 (95% CI: 1.03−2.58) and 1.31 (95% CI: 0.97−1.77), respectively. The overall weighted PFS HR was 0.70 (95% CI: 0.59−0.84). The efficacy of elotuzumab triplet regimens did not significantly differ from that of non-elotuzumab-based regimens. Elotuzumab triplet regimens had better efficacy profiles (ORR and at least VGPR) compared to doublet and single regimens. Single regimens had little efficacy in RRMM patients. These meta-analysis results indicated elotuzumab triplet regimens were more effective than non-mAb-based regimens based on the ORR, at least VGPR, and PFS.

#### Efficacy of daratumumab-based regimens

**Figure 4:**
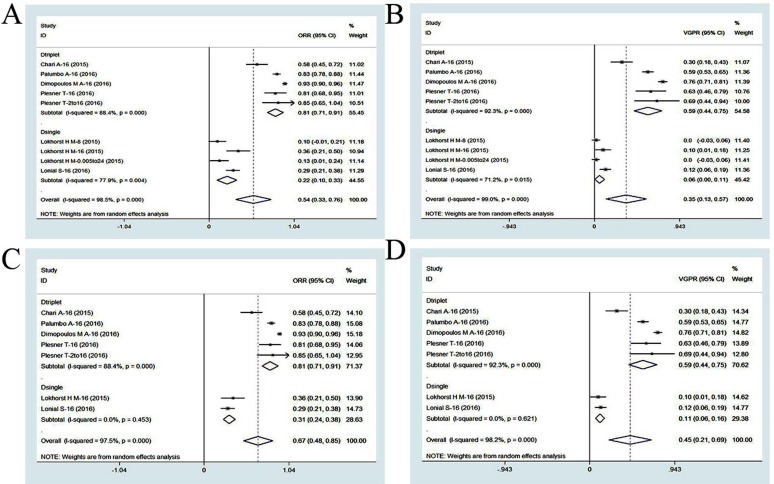
Meta-analysis of the efficacy of daratumumab-based regimens in patients with RRMM:(A) overall response rate of daratumumab-based single and triplet regimens;(B) at least very good partial response of daratumumab-based single and triplet regimens;(C) overall response rate of daratumumab-based monotherapy (16mg/kg);(D) at least very good partial response of daratumumab-based monotherapy (16mg/kg) ORR, overall response rate; VGPR, very good partial response;CI, confidence interval.Dtriplet, daratumumab-based triplet regimen; Dsingle, daratumumab-based single regimen.

There were six studies (869 patients) that evaluated the efficacy of daratumumab for the treatment of RRMM based on the ORR and at least VGPR [5, 12, 13, 33–35]. The ORR was 54% (95% CI: 33−76%) and the at least VGPR was 35% (95% CI: 13−57%) in a pooled analysis (Figure 4A-4B). In a subgroup analysis (triplet/single), the ORR was 81% (95% CI: 71−91%) for triplet regimens compared to 22% (95% CI: 10−33%) for single regimens, indicating the triplet regimens were more effective. The same trend was observed in the at least VGPR. The at least VGPR was 59% (95% CI: 44−75%) for triplet regimens and 6% (95% CI: 0−11%) for single regimens, indicating triplet regimens were more favorable. Since 16 mg/kg is the approved dosage of daratumumab [11, 12], two clinical trials were excluded from our analysis (daratumumab single regimens with non-16 mg/kg doses). A pooled analysis after exclusion showed that the ORR and at least VGPR of a daratumumab-based single regimen (16 mg/kg) were 31% (95% CI: 24−38%) and 11% (95% CI: 6−16%), respectively. Notably, all enrolled patients in this daratumumab-based single regimen subgroup analysis were heavily pretreated MM patients with highly refractory disease. Interestingly, elotuzumab had no effect in heavily pretreated MM patients, which highlighted the effectiveness of daratumumab (Figure 4C-4D) [29].

We next analyzed the ORs and HRs reported in two head-to-head RCTs (1,067 patients) that evaluated daratumumab triplet regimens (Figure 2C-2E) [5, 13]. The overall weighted ORs of the ORR and at least VGPR were 3.25 (95% CI: 2.31−4.56) and 3.75 (95% CI: 2.88−4.88), respectively, while the PFS HR was 0.38 (95% CI: 0.30−0.48) for daratumumab-based triplet regimens compared to non-daratumumab-based regimens. These results indicated that daratumumab-based triplet regimens had favorable effects on ORR and VGPR compared to single regimens, although single regimens at the optimal dosage were also effective. Daratumumab-based triplet regimens were more effective than non-daratumumab-based regimens according to the ORR, at least VGPR, and PFS. Daratumumab monotherapy (16 mg/kg) has a remarkable efficacy profile for heavily pretreated MM patients. A summary of patient responses and survival outcomes is shown in Table 3.

**Table 3:** Summary of response and survival outcomes from mAbs

Regimen	No. of trials	ORR (95% CI)	*P* for ORR	At least VGPR(95% CI)	*P* for at least VGPR	OR of ORR (95% CI)	OR of at least VGPR (95% CI)	HR of PFS (95% CI)
**mAb**	13	57(38-76)		32(19-46)				
Triplet	10	76(69-84)	P_23_<0.000	48(34-61)	P_23_<0.000	2.3(1.48-3.56)	2.33(1.25-4.33)	0.52(0.36-0.75)
Doublet	1	48(29-67)	P_12_=0.002	7(-2-17)	P_12_=1			
Single	2	17(4-31)	P_13_<0.000	4(0-8)	P_13_<0.000			
**Elotuzumab**	7	60(29-91)		29(15-44)				
Triplet	5	73(61-84)	P_23_<0.000	38(27-48)	P_23_=0.002	1.63(1.03-2.58)	1.33(0.97-1.77)	0.70(0.59-0.84)
Doublet	1	48(29-67)	P_12_<0.000	7(-2-17)	P_12_=0.192			
Single	1	1(-3-6)	P_13_<0.000	1(-3-6)	P_13_<0.000			
**Daratumumab**	6	54(33-76)		35(13-57)				
Triplet	4	81(71-91)	Pd<0.000	59(44-75)	Pd<0.000	3.25(2.31-4.56)	3.75(2.88-4.88)	0.38(0.30-0.48)
Single(16mg/kg)	2	31(24-38)		11(6-16)				

mAb: monoclonal antibody; OR: odds ratio; ORR: overall response ratio; VGPR: very good partial response; HR: hazard ratio; PFS: progression-free survival. P_23_, P_12_, P_13_: The value between triplet and doublet, single and doublet, single and triplet, respectively. And when the P_23_, P_12_, or P_23_ is less than 0.0125, the efficacy of ORR or at least VGPR between the group is significantly. Besides, when the Pd value is less than 0.05, the efficacy of ORR or at least VGPR between the group is significantly.

### Safety

We performed a pooled analysis to analyze the rate ratio of grade 3/4 adverse events in all included trials. The most frequent hematological adverse events were neutropenia (30%, 95% CI: 17–43%), lymphopenia (24%, 95% CI: 0–49%), and thrombocytopenia (17%, 95% CI: 10–23%). The most common non-hematological adverse events included pneumonia (8%, 95% CI: 6–10%) and fatigue (5%, 95% CI: 3–7%) (Table 4A). We also performed a meta-analysis to analyze the risk ratios of grade 3/4 adverse events in four RCTs that compared mAb- and non-mAb-based regimens (Table 4B). No differences in most grade 3/4 adverse events were detected with the exception of lymphopenia (risk ratio: 1.83, 95% CI: 1.16–2.87, P = 0.009), and diarrhea (risk ratio: 1.61, 95% CI: 1.01–2.56, P = 0.046). We performed a subgroup analysis (Table 4) to investigate the most common hematological and non-hematological adverse events resulting from daratumumab and elotuzumab treatment. The most frequent hematological adverse events were neutropenia, lymphopenia, and thrombocytopenia. Elotuzmumab-based regimens primarily resulted in lymphopenia and neutropenia, whereas daratumumab-based regimens primarily caused neutropenia and thrombocytopenia. No statistically significant differences were observed with the addition of mAbs to the treatment regimen, with the exception of increased lymphopenia and diarrhea. The same trend was observed after the addition of elotuzumab, with the exception of an increase in lymphopenia. An unexpected decrease in the occurrence of neutropenia was observed with the addition of elotuzumab to the treatment regimens. This may be one advantage of elotuzumab. Additional studies with larger patient populations are necessary to confirm the benefits of elotuzumab. There were no additional grade 3/4 adverse events observed with daratumumab-based regimens.

**Table 4A:** Pooled analysis of common grade at least 3 adverse events

Adverse event	No. of trials	Rate ratio in E. (%,95%CI)	Rate ratio in D. (%,95%CI)	Rate ratio in mAbs (%,95%CI)
**Hematologic**				
Neutropenia	10[5, 11–13, 27, 28, 30, 33–35]	25(13,38)	34(15,53)	30(17,43)
Lymphopenia	6[5, 11, 13, 27, 30, 33]	41(-2,84)	7(4,11)	24(0,49)
Thrombocytopenia	11[5, 11–13, 27, 28, 30, 32–35]	16(11,20)	18(6,29)	17(10,23)
Anemia	11[5, 11–13, 27–30, 32–35]	11(5,18)	14(8,20)	13(9,17)
Leukopenia	4[27, 30, 33, 35]	9(3,14)	9(-4,21)	8(2,14)
**Nonhematologic**				
Pneumonia	5[5, 13, 28, 30, 35]	9(1,16)	8(6,10)	8(6,10)
Fatigue	11[5, 11–13, 27, 28, 30, 32–35]	7(5,10)	4(2,6)	5(3,7)
Peripheral neuropathy	5[5, 27, 30–32]	7(2,11)	5(2,7)	5(3,7)
Diarrhoa	10[5, 11, 13, 27, 28, 30, 32–35]	6(4,8)	3(2,5)	4(3,6)
Pyrexia	11[5, 11, 13, 27, 28, 30–35]	3(1,4)	1(1,2)	2(1,2)
Back pain	7[11, 13, 27, 28, 31, 33, 34]	3(-1,6)	2(1,3)	2(0,3)

**Table 4B:** Meta-analysis of mAb-based therapies common grade at least 3 adverse events

Adverse event	No. of trials	Events in mAb arm	Events in control arm	Risk ratio(95% CI)	*p*
**Hematologic**					
Neutropenia	3[5, 11, 13]	285/844	252/835	1.36(0.77, 2.41)	0.293
Lymphopenia	3[5, 11, 13]	282/844	170/835	1.83(1.16, 2.87)	0.009*
Thrombocytopenia	4[5, 11, 13, 32]	214/919	193/910	1.02(0.75, 1.39)	0.886
Anemia	4[5, 11, 13, 32]	135/919	165/910	0.81(0.66, 1)	0.051
**Nonhematologic**					
Pneumonia	2[5, 13]	42/526	46/518	0.90(0.6, 1.34)	0.600
Fatigue	4[5, 11, 13, 32]	59/919	42/910	1.42(0.89, 2.26)	0.137
Peripheral Neuropathy	2[5, 32]	18/318	25/312	0.71(0.4, 1.27)	0.251
Diarrhoa	4[5, 11, 13, 32]	46/919	28/910	1.61(1.01, 2.56)	0.046*
Pyrexia	4[5, 11, 13, 32]	16/919	19/910	0.90(0.46, 1.75)	0.749
Insomnia	4[5, 11, 13, 32]	8/919	14/910	0.63(0.27, 1.50)	0.298

**Table 4C:** Meta-analysis of daratumumab-based therapies common grade at least 3 adverse events

Adverse event	No. of trials	Events in D. arm	Events in control arm	Risk ratio(95% CI)	*p*
**Hematologic**					
Neutropenia	2[5, 13]	178/526	114/518	1.918(0.90, 4.09)	0.092
Lymphopenia	2[5, 13]	38/526	16/518	2.31(0.93, 5.70)	0.07
Thrombocytopenia	2[5, 13]	146/526	116/518	1.82(0.82, 1.71)	0.358
Anemia	2[5, 13]	70/526	93/518	0.746(0.53, 1.05)	0.095
**Nonhematologic**					
Pneumonia	2[5, 13]	42/526	46/518	0.898(0.60, 1.34)	0.600
Fatigue	2[5, 13]	29/526	15/518	1.87(0.997, 3.52)	0.051
Peripheral neuropathy	1[5]	11/243	16/237	0.671(0.32, 1.42)	0.294
Diarrhoa	2[5, 13]	24/526	12/518	1.943(0.98, 3.86)	0.058

**Table 4D:** Meta-analysis of elotuzumab-based therapies common grade at least 3 adverse events

Adverse event	No. of trials	Events in E. arm	Events in control arm	Risk ratio(95% CI)	*p*
**Hematologic**					
Neutropenia	1[11]	107/318	138/317	0.773(0.63, 0.94)	0.011*
Lymphopenia	1[11]	244/318	154/317	1.579(1.39, 1.80)	0.00*
Thrombocytopenia	2[11, 32]	68/393	77/392	0.829(0.51, 1.34)	0.439
Anemia	2[11, 32]	65/393	72/392	0.899(0.66, 1.22)	0.49
**Non-hematologic**					
Fatigue	2[11, 32]	30/393	27/392	1.092(0.66, 1.81)	0.731
Peripheral neuropathy	1[32]	7/75	9/75	0.778(0.31, 1.98)	0.598
Diarrhoa	2[11, 32]	22/393	16/392	1.366(0.13, 2.57)	0.333
Insomnia	2[11, 32]	7/393	9/392	0.776(0.30, 2.06)	0.611
Pyrexia	2[11, 32]	8/393	12/392	0.609(0.14, 2.65)	0.508

mAb: monoclonal antibody; D.:daratumumab; E.:elotuzumab; *: significantly difference between groups in adverse events(*P* < 0.05).

### Infusion-related reactions

**Figure 5:**
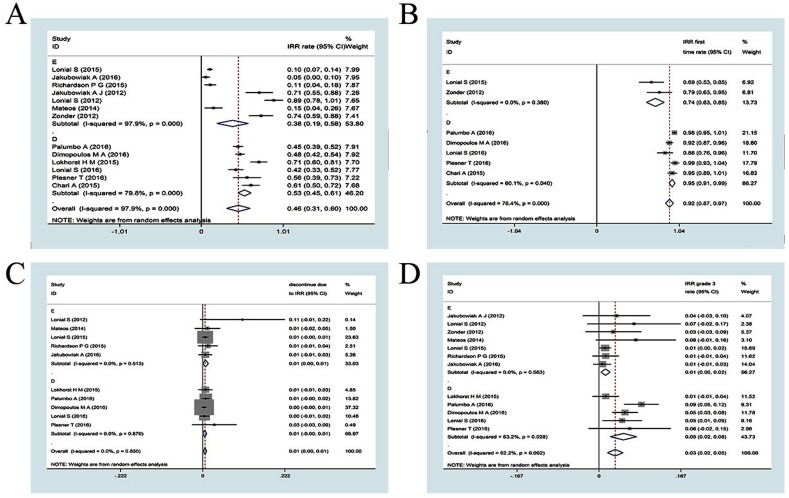
Meta-analysis of the IRRs of mAbs-based regimens in patients with RRMM: (A) any grade infusion-related reactions rate of mAbs;(B) the rate of IRR occurs in first time infusion; (C) grade 3 infusion-related reactions rate of mAbs;(D) the rate of discontinue due to IRRs IRR, infusion related reactions; CI, confidence interval; E: elotuzumab; D: daratumumab

Based on the pooled analysis of the clinical trials included in our study, infusion-related reactions (any grade) were observed in 46% (95% CI: 31–60%) of the patients, and infusion-related reactions (at least grade 3) were observed in only 3% (95% CI: 2–5%) of patients. The infusion-related reactions were primarily observed during the first infusion (92%, 95% CI: 87–97%). The rate of patients who discontinued the trial due to infusion-related reactions was also very low (1%, 95% CI: 0–1%). In elotuzumab-based clinical trials, infusion-related reactions (any grade) were observed in 38% (95% CI: 19–58%) of patients, while infusion-related reactions (at least grade 3) were observed in only 1% (95% CI: 0–2%) of patients. The infusion related reactions primarily occurred during the first infusion (74%, 95% CI: 63–85%). The rate of patients who discontinued due to infusion-related reactions was also very low (1%, 95% CI: 0–1%). In daratumumab-based trials, infusion-related reactions (any grade) were observed in 53% (95% CI: 45–61%) of patients, while infusion-related reactions (at least grade 3) were observed in 5% (95% CI: 2–8%) of patients. The infusion-related reactions were primarily observed during the first infusion (95%, 95% CI: 91–99%). The rate of patients who discontinued due to infusion-related reactions was also very low (1%, 95% CI: 0–1%) (Figure 5A-5D). Collectively, the results indicated that patients treated with mAb-based regimens suffered infusion-related reactions that predominantly occurred during the first infusion, resulting in some patients discontinuing the trials. These reactions were more frequent among patients treated with daratumumab than elotuzumab.

### Sensitivity analysis

**Figure 6:**
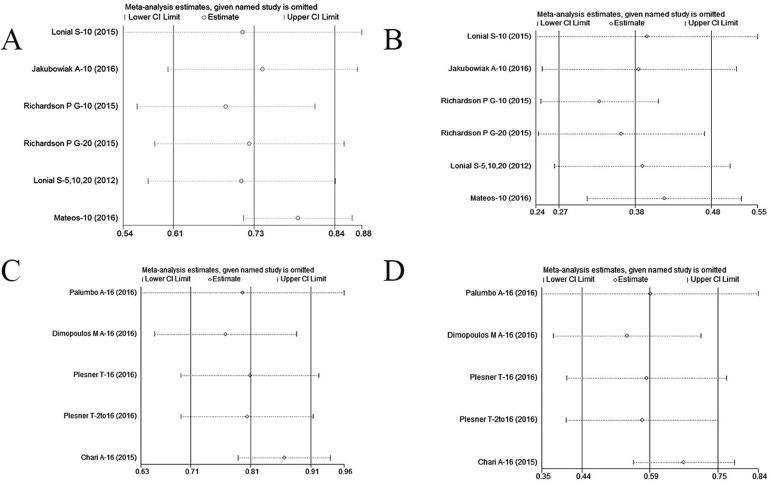
Leave-one-out analysis of the efficacy of daratumumab and elotuzumab-based triplet regimens in patients with RRMM: (A) overall response rate of elotuzumab-based triplet regimens;(B) at least very good partial response of elotuzumab-based triplet regimens;(C) overall response rate of daratumumab-based triplet regimens;(D) at least very good partial response of daratumumab-based triplet regimens CI, confidence interval.

We performed a sensitivity analysis of daratumumab and elotuzumab-based triplet regimens using the leave-one-out method in patients with RRMM (Figure 6A-6D). The results indicated that two clinical trials significantly influenced results of the pooled analysis [31, 33] (Figure 6A-6D). These trials involved elotuzumab in combination with thalidomide and dexamethasone [31], and daratumumab in combination with pomalidomide and dexamethasone [33]. This analysis suggested that a mAb in combination with either thalidomide or pomalidomide is not more effective than a mAb in combination with either lenalidomide or bortezomib.

## DISCUSSION

### The efficacy and safety of mAbs for the treatment of RRMM

In our aggregated analysis, the efficacy and safety trends were reinforced in the pooled population. Our data indicate mAb-based therapy is a superior alternative to non-mAb-based therapy because it improves the ORR, at least VGPR, and PFS in RRMM patients, particularly when triplet combination regimens are utilized. Subgroup analysis indicated that mAb-based triplet regimens were superior to doublet regimens, and that doublet regimens were more effective than single regimens. The same trend was also observed for both daratumumab- and elotuzumab-based regimens (Table 3).

Minimal toxicities were associated with the addition of mAbs to the therapeutic regimen. Infusion-related reactions were the most commonly reported adverse events and primarily occurred during the first infusion. We found that they occurred more frequently in daratumumab- than elotuzumab-based clinical trials. Adequate and timely management of infusion-related reactions is important in order to prevent toxicity and treatment discontinuation. If patients experience infusion-related reactions, the infusion should be temporarily interrupted, and the patients should be treated with glucocorticoids or antihistamines at the discretion of the physician [36].

Daratumumab and other CD38-targeting antibodies can interfere with blood typing by binding to CD38 on the surface of red blood cells (RBCs) and leading to a positive indirect Coombs test. Daratumumab interference in pre-transfusion tests can be negated by denaturation of surface CD38 on RBCs using the reducing agent dithiothreitol [37]. Patients should undergo extensive RBC antigen phenotyping and screening prior to receiving the first infusion of daratumumab or any other CD38-targeting antibody [38]. Our results demonstrate that novel mAb-based regimens achieve superior responses compared to non-mAb-based regimens, without a risk of toxicity. We performed cross-trial comparisons of newly marketed agents in different combinations in order to provide more practical suggestions for clinical decision-making.

### The best option among novel agent-based triplet regimens for RRMM

Despite confounding caused by different study designs and populations, we conducted a cross-trial comparative analysis to determine the best option among the novel agent-based triplet regimens for RRMM treatment. We ranked the regimens based on the efficacy profiles in the RCTs that investigated the combination of a novel agent and traditional doublet therapy (Table 5). A total of five novel agent-based triplet regimens were evaluated. The daratumumab-based triplet regimen was expected to be the most potent of the regimens.

**Table 5:** Summary of response and survival outcomes from novel agent-based regimens

Novel agent	Regimen	Median prior therapy (range)	ORR (%)	At least VGPR (%)	HR of PFS	Median PFS (months)
**Triplet**						
Daratumumab [13]	D+R+d/R+d	1(1-11)/1(1-8)	92.9/76.4	75.8/44.2	0.37	NR/18.4
Daratumumab [5]	D+V+d/V+d	2(1-9)/2(1-10)	82.9/63.2	59.2/29	0.39	NR/7.2
Carfilzomib [8]	C+R+d/R+d	2(1-3)	87.1/66.7	69.9/40.4	0.69	26.3/17.6
Elotuzumab [11]	E+R+d/R+d	2(1-4)	78.5/65.5	32.7/28	0.70	19.4/14.9
Elotuzumab [32]	E+V+d/V+d	1(1-3)	66.2/62.6	36.3/26.7	0.72	9.7/6.9
Ixazomib [10]	I+R+d/R+d	1(1-3)	78.3/71.5	48.1/39	0.74	20.6/14.7
Panobinostat [9]	P+V+d/V+d	1(1-3)	60.7/54.6	27.6/15.7	0.63	12/8.1
**Doublet**						
Carfilzomib [39]	C+d/V+d	2(1-3)	76.7/62.4	54/29	0.53	18.7/9.4
Elotuzumab [30]	E+V	2(1-3)	48.1	7.4	NE	9.46
Pomalidomide [7]	Po+d/d	5(2-14)/5(2-17)	31/10	4.6/0.6	0.48	4/1.9
**Single**						
Daratumumab-16	D	5(2-14)	31	1	NE	4
Carfilzomib [40]	C	5(1-20)	28	10	NE	NE
Pomalidomide [40]	Po	5(1-17)	19	2	NE	NE
Elotuzuamb [29]	E	4.5(2-10)	0	0	NE	NE

E: elotuzumab; D: daratumumab; C: carfilzomib; I: ixazomib; P: panobinostat; Po: pomalidomide; R: lenalidomide; V: bortezomib; d: dexamethasone. Daratumumab-16: daratumumab with optimal dosage of 16 mg/kg; ORR: overall response rate; VGPR: very good partial response; NR: not reach; NE: not estimated.

We found that lenalidomide-based regimens were superior to bortezomib, thalidomide, or pomalidomide-based triplet regimens since the efficacy of daratumumab in combination with lenalidomide plus dexamethasone was superior to daratumumab in combination with dexamethasone plus either pomalidomide or bortezomib. Similarly, the efficacy of elotuzumab in combination with lenalidomide plus dexamethasone was superior to elotuzumab in combination with dexamethasone plus either bortezomib or thalidomide (Table 5 and Figure 6). Thus, daratumumab in combination with lenalidomide plus dexamethasone is currently the best option among all triplet regimens for RRMM.

### The best option among novel agent-based doublet regimens for RRMM

A similar cross-trial comparison was performed to identify the best option among the novel agent-based doublet regimens. We evaluated three different novel agent-based doublet regimens. The regimens were ranked based on the efficacy profiles (Table 5). Daratumumab was not evaluated because there was a lack of data for doublet regimens. Among the doublet agent-based regimens, traditional agents such as bortezomib or lenalidomide plus dexamethasone showed reasonable efficacy. Additional head-to-head clinical trials are required to compare the efficacy between traditional agent-based doublet regimens to novel agent-based doublet regimens. Currently, the best option among novel agent-based doublet regimens is carfilzomib plus dexamethasone.

### The best option among novel agent-based single regimens for RRMM

The meta-analysis results demonstrated that daratumumab monotherapy (16 mg/kg dose) yielded an ORR of 31.1% and at least VGPR of 11.5%. It was therefore ranked first among novel single agents. Since all of the trials analyzed included heavily treated MM patients, daratumumab at a dose of 16 mg/kg was superior to the other novel single agents among these patients. In contrast, elotuzuamb monotherapy had no effect in these patients. A summary of the responses and survival outcomes of patients treated with the novel agent-based regimens is shown in Table 5.

### Limitations and outlook

Our meta-analysis had several limitations including high study heterogeneity. Although all clinical trials were talked about the response and adverse events of elotuzumab or daratumumab, the inclusion criteria of the clinical trials included in the meta-analysis significantly differed. Second, long-term observation and follow-up is needed in order to analyze overall survival (OS) and confirm the efficacy and safety of the various regimens. Thirdly, language bias may occur because of language barriers as we only searched among English publications. Finally, the results of the cross-trial comparison may be confounded by differences in study design and context. Additional data from head-to-head comparisons between the various triplet regimens is required to inform clinical decision-making [41]. RCTs and follow-up studies are ongoing and are necessary to validate our findings.

mAb-based therapy is a critical strategy for cancer treatment. It is aimed at engaging or augmenting the immune system to target cancer cells [42]. Our results indicate that triplet regimens that include a mAb, particularly daratumumab, are more effective than doublet and single regimens in RRMM. However, triplet regimens are associated with higher costs. Therefore, further analysis of the cost-effectiveness of triplet and doublet regimens is highly recommended. We demonstrated that both novel mAbs had reasonable efficacy and safety profiles. Daratumumab was highly effective in both triplet and single regimens in both general and heavily pretreated MM patients. However, possible infusion-related reactions, interference with blood tests, and drug resistance should be considered when treating patients with daratumumab.

The effects of daratumumab suggests that CD38 may be a prominent therapeutic target. Interestingly, two new agents, MOR202 (MOR03087) and isatuximab (SAR650984), which target CD38, have already demonstrated favorable efficacy and safety in the clinic. MOR202 has been evaluated as monotherapy and in combination with either pomalidomide or lenalidomide in ongoing phase I/IIa clinical trials (NCT01421186). A positive ORR and VGPR were observed with MOR202 monotherapy in approximately 31% and 13% of patients, respectively. The duration of MOR202 infusion is 2 hours, which is relatively brief in comparison to daratumumab and the rate of infusion-related reactions is low (10% compared to 48% with daratumumab [43]. Isatuximab demonstrated significant activity as a single agent in a phase I trial involving 35 patients with RRMM who had received a median of six prior lines of therapy. At doses above 10 mg/kg, the ORR and VGPR were 33% and 11%, respectively [44]. It also demonstrated clear activity as a single agent in a phase II trial involving 97 patients with RRMM who had received a median of five prior lines of therapy resulting in an ORR of 24%. Infusion-related reactions occurred in 49% of the patients [45]. Since both mAbs have promising efficacy and safety profiles, they may be particularly useful for RRMM treatment, especially in heavily pretreated patients.

Daratumumab exerts its anti-cancer effects through promoting cellular cytotoxicity [16, 17], phagocytosis [17, 18], and induction of apoptosis [17, 19]. Besides, according to the recent study, it also depletes immunosuppressive regulatory T cells, B cells, and myeloid-derived suppressor cells that are CD38-positive [20]. Cytotoxic T-cell number, activation and clonal expansion increased after daratumumab treatment in heavily pretreated MM patients [20]. Thus, the mechanism of anti-CD38 novel agent may suggest that they possess an outstanding effect on tumor cells than other traditional regimens in triplet regimens for RRMM and single regimen for heavily pretreated MM patients.

The unique mechanism of action of daratumumab and the minimal toxicity of this agent suggest that it may be the most effective of these agents for RRMM treatment. The treatment against MM is continuously evolving due to the development of new mAbs, especially CD38 and PD1 [46, 47] (Table 6). Future clinical studies of these new agents will reveal which combinations are the most effective for maintenance therapy, newly diagnosed multiple myeloma, and RRMM [46].

**Table 6:** Monoclonal antibodies being evaluated in multiple myeloma

Antibody	Target	Phase
Isatuximab (SAR650984)	CD38	III
MOR202	CD38	I/IIa
Milatuzumab	CD74	I/II
Indatuximab ravtansine (drug conjugate)	CD138	I/II
Tabalumab	B-cell activating factor	II
Siltuximab	IL6	II
Lucatumumab	CD40	I
Dacetumumab	CD40	I
BHQ880	DKK1	II
Sotatercept (RAP-011)	Activin receptor ligand trap	IIa
huN901-DM1 (drug conjugate)	CD56	I
Pembrolizumab	PD1	II/III
Nivolumab	PD1	II/III
Atezolizumab	CD274 (PD-L1)	I

IL6, interleukin 6; PD1, programmed cell death 1; PD-L1, programmed cell death ligand 1.

## MATERIALS AND METHODS

### Search strategy

This study was performed in accordance with the preferred reporting items for systematic reviews and meta-analyses (PRISMA) statement [48, 49]. We queried the EMBASE, MEDLINE, Cochrane Library, Clinicaltrials.gov, American Society of Hematology, American Society of Clinical Oncology, and European Hematology Association databases to identify clinical trials that investigated the outcomes of patients who received elotuzumab- and daratumumab-based therapy for RRMM. The following medical terms were used in the search: (1) daratumumab, (2) darzalex, (3) elotuzumab, (4) empliciti, (5) 1 OR 2 OR 3 OR 4, (6) relapsed, (7) refractory, (8) 6 OR 7, (9) myeloma, and (10) 5 AND 8 AND 9 in the study titles or abstracts. The search was limited to publications in English. The reference lists of included articles were manually reviewed to identify eligible studies that may have been missed during the initial search. The search results were last updated in November 2016.

### Study selection and endpoints

Studies were eligible for inclusion in the meta-analysis if they met all the following criteria: (1) publication date between January 2005 and November 2016; (2) clinical trial; (3) intervention group containing elotuzumab or daratumumab; (4) investigated patients with RRMM; and (5) at least one of the following data was reported: ORR, VGPR, PFS, or OS. The article with the most recent publication date was selected if multiple publications were available for a given study. All potentially relevant articles were reviewed by two independent investigators (Tiantian Zhang and Sen Wang). Discrepancies in study eligibility were resolved through discussions among investigators. Our primary efficacy endpoints of interest were ORR and at least VGPR, and the other important endpoints were survival (PFS and OS). The safety endpoints were grade 3/4 treatment-related adverse events and infusion-related reactions.

### Data extraction

Study data was collected by two independent reviewers (Tiantian Zhang and Sen Wang), and included the name of the first author, year of publication, trial name, clinical trial phase, median patient age, therapeutic regimen, the median number of prior therapies, number of patients, median follow-up time, median PFS, and OS.

### Statistical analysis

Because there were some phase I and II clinical trials that did not have control arms, we performed a pooled analysis for all clinical trials and a meta-analysis of RCTs [23, 40]. Rate ratios and 95% CIs were used to describe clinical outcomes (efficacy and safety) in a single arm according to the ORR and at least VGPR, and adverse events. ORs and 95% CIs were used to describe clinical outcomes (efficacy) according to the ORR and at least VGPR. PFS HRs and 95% CIs for the intervention versus control arm were representative of treatment efficacy. The RR and associated 95% CI were used to describe safety based on the effect size.

The meta-analysis was performed using Stata (v14). A random effects model was utilized to calculate the pooled HR, ratio rate, OR, and 95% CI. Inter-study heterogeneity was estimated using Cochran's Q and I^2^ tests. A P value < 0.1 or an I^2^ statistic > 50% was indicative of significant heterogeneity. A random-effects model was selected if the I^2^ was significant, and a fixed-effects model was selected in all other cases. Clinical trials were classified into subgroups according to the therapeutic regimen: elotuzuamb- or daratumumab-based triplet, doublet, or single-agent. Sensitivity analysis was performed by sequentially excluding individual studies and recalculating the ORR and at least VGPR. Comparisons between three groups were performed with partitions of Pearson's chi-square statistic or Fisher's exact test. Comparisons between two groups were performed using Pearson's chi-square or Fisher's exact tests for categorical variables. Forest and leave-one-out sensitivity analysis plots were generated with Stata (v14). All tests were two-sided and a P < 0.05 was considered statistically significant.

## Abbreviations

mAb: Monoclonal antibody
RRMM: relapsed or relapsed and refractory multiple myeloma
RCT: randomized controlled trial
ORR: overall response rate
VGPR: very good partial response
PI: proteasome inhibitors
IMiD: immunomodulatory
NR: not reach
NE: not estimated
HR: hazard ratio
RR: risk ratio
OR: odds ratio
AE: adverse event
IRR: infusion related reaction
PFS: progression-free survival
OS: overall survival
HDAC- inhibitor: histone deacetylase inhibitor
SLAMF7: signaling lymphocytic activation molecule F7
NA: not available or no target points
